# Proteomic and metabolomic analyses provide insight into the off-flavour of fruits from citrus trees infected with ‘*Candidatus* Liberibacter asiaticus’

**DOI:** 10.1038/s41438-018-0109-z

**Published:** 2019-02-14

**Authors:** Lixiao Yao, Qibin Yu, Ming Huang, Weilun Hung, Jude Grosser, Shanchun Chen, Yu Wang, Frederick G. Gmitter

**Affiliations:** 1 Citrus Research Institute, Southwest University, Chongqing, China; 2 Citrus Research and Education Center, University of Florida, Lake Alfred, FL 33850 USA

**Keywords:** Biotic, Systems analysis

## Abstract

Orange fruit from trees infected by ‘*Candidatus* Liberibacter asiaticus’ (*Ca*Las) often do not look fully mature and exhibit off-flavours described as bitter, harsh, and metallic rather than juicy and fruity. Although previous studies have been carried out to understand the effect of *Ca*Las on the flavour of orange juice using metabolomic methods, the mechanisms leading to the off-flavour that occurs in Huanglongbing (HLB)-symptomatic fruit are not well understood. In this study, fruits were collected from symptomatic and healthy Valencia sweet orange (*Citrus sinensis*) trees grafted on Swingle (*C. paradisi* X *Poncirus trifoliata*) rootstock. Isobaric tags for relative and absolute quantification (iTRAQ) and gas chromatography-mass spectrometry (GC-MS) were used to measure the proteins, sugars, organic acids, amino acids, and volatile terpenoids. The results showed that most of the differentially expressed proteins involved in glycolysis, the tricarboxylic acid (TCA) cycle and amino-acid biosynthesis were degraded, and terpenoid metabolism was significantly downregulated in the symptomatic fruit. Valencene, limonene, 3-carene, linalool, myrcene, and α-terpineol levels were significantly lower in fruit from *Ca*Las-infected trees than from healthy trees. Similar phenomena were observed for sucrose and glucose. Our study indicated that off-flavour of symptomatic fruit was associated with a reduction in the levels of terpenoid products and the downregulation of proteins in glycolysis, the TCA cycle, and the terpenoid biosynthesis pathway.

## Pathogenic profiling: why fruit from bacteria-infected trees tastes bad

The bacteria *Candidatus* Liberibacter asiaticus (*Ca*Las) causes orange trees to produce poor-tasting fruit thanks to the decreased production of flavour-enhancing proteins, sugars, and metabolites. The University of Florida’s Frederick Gmitter and his team of US and Chinese scientists profiled the proteins and metabolites of healthy Valencia sweet orange trees infected with CaLas, a bacterial pathogen that causes the citrus disease Huanglongbing and reduces the quantity and quality of fruit and juice. The researchers found 123 differentially-expressed proteins and decreased numbers of taste-enhancing constituents, including those mediated by key energy-producing processes. This degradation involved a class of chemicals called terpenoids, which the authors link to poor quality fruit. These results provide insights into the pathogenesis of CaLas infection and could empower future studies to prevent the impact of the bacteria and Huanglongbing infection.

## Introduction

In Florida, the production of sweet oranges dropped from 240 million boxes in 2004 to 45 million in 2018, substantially because of the most devastating citrus disease named Huanglongbing (HLB) (Florida Citrus Commission: http://www.floridacitrus.org). ‘*Candidatus* Liberibacter asiaticus’ (*Ca*Las) is the main pathogen that induces HLB and is naturally transmitted via the Asian citrus psyllid, *Diaphorina citri*, or by grafting with infected scions. *Ca*Las can be present in leaves, bark, roots, flowers, and fruit, and the leaf midribs are usually used for detecting the pathogen^1,2^.

HLB not only reduces the quantity of citrus fruit but also affects the quality of fruit and orange juice. HLB-impacted fruits are small, asymmetrical, and lopsided, and have small or aborted seeds. The axis of symptomatic fruit becomes bent, and the central vascular bundles in fruit become deep yellow-orange to brown^3^.The fruit surfaces can be substantially green even at fruit maturity, and may ‘break’ only on the stem end. Hence, HLB is also called ‘citrus greening’. The juice of HLB-impacted mature fruit is lower in percentage and soluble solid content, higher in acidity, has a bitter, harsh, metallic taste, and numerous off-flavours^4^.

In an attempt to reveal how the flavour of HLB orange juice is changed, the chemical compound composition of diseased fruit were examined by several research groups^5–9^. Lower amounts of sugars and higher amounts of titratable acid are found in the HLB-affected fruits, resulting in a lower soluble solid/acid ratio^5–7^. A lower soluble solid/acid ratio often indicates that the symptomatic fruits are bitter and sour and similar to an unripe product. Bitter principles, such as limonin, are elevated greatly in juice from the symptomatic fruit. However, none of the limonin levels observed exceed those of the average bitterness threshold in HLB juice^5,8,9^. The investigation of orange juice from *Ca*Las-infected and *Ca*Las-free trees by gas chromatography-mass spectrometry (GC-MS) showed that symptomatic Valencia orange juice has similar total volatiles to those of the healthy control juice but only 40% of the total esters, 48% of the total aldehydes, and 33% of the total sesquiterpenes of the control juice^5^. The concentration of valencene is also 50–67% lower in juice from symptomatic fruit compared with that of the healthy control. The levels of esters, especially ethyl butanoate and ethyl 2-methylbutyrate, and soapy-waxy alkanals, such as octanal and decanal, are significantly lower in HLB-impacted orange juice^5,8^, whereas the levels of juice terpenes (such as γ-terpinene and α-terpinolene), alcohol, green aldehydes (such as hexanal), and degradation compounds of limonene and linalool (such as α-terpineol) are higher in symptomatic juice than those in the control^8^. Therefore, the previously reported off-flavour associated with HLB-symptomatic juices is likely not bitterness alone but is apparently the result of the juices being less sweet and more sour due to lower sugar and higher acid levels and an imbalance of certain volatile compounds^6,8,9^.

Research has been conducted to identify key genes, proteins, and organic products induced by *Ca*Las in leaf tissues through transcriptomic, proteomic, or metabolomic studies^10–14^. These studies have shown that the vital biological pathways and processes, such as cell defence, transport, photosynthesis, carbohydrate metabolism, and hormone metabolism, are affected by the disease. HLB-impacted fruit has a modified carbohydrate and phytohormone balance^15^. Two research teams analysed the whole-genome expression of fruit peel from *Ca*Las-infected trees. They found that the transcription of genes involved in the light reactions of photosynthesis and in ATP synthesis was enhanced, and the proteins involved in degradation and misfolding processes were activated under the HLB stress. At the same time, HLB also strongly affected source-to-sink communication pathways, including sucrose and starch metabolism and hormone synthesis and signalling^1,16^.

Proteomics and metabolomics, each with its own advantages, have different analytical objects. Proteomics aims to detail an overall and comprehensive understanding of protein characteristics directly relevant to physiological phenotypes and underlying processes. Metabolomics aims to analyse quantitatively all low molecular weight biomolecules that are the end products of reactions mediated by proteins during a specific physiological period. The integration of proteomic and metabolomic analyses in one study could create a more comprehensive overview of changes in protein expression and metabolite composition and aid the understanding of the metabolic and physiological changes of HLB-affected fruits. In this study, we used isobaric tags for relative and absolute quantification (iTRAQ) and GC-MS to measure the proteins and metabolic compounds of the juice sacs from symptomatic and healthy fruits. The relationship between metabolic compounds and differentially expressed proteins in pulp of HLB-affected and healthy fruit was assessed, revealing the molecular mechanisms determining off-flavour in symptomatic fruits.

## Results

### *Ca*Las detection and HLB-affected fruit symptoms

**Fig. 1:**
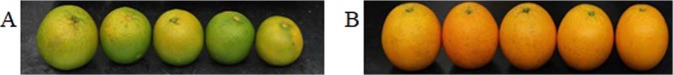
HLB-symptomatic (**A**) and healthy (**B**) fruits from Valencia grafted on Swingle rootstock

Healthy and *Ca*Las-infected trees were selected from a commercial citrus grove and confirmed by quantitative PCR (qPCR) with *Ca*Las-specific primers^17^. The leaves from healthy trees were PCR negative (Ct > 32) and those from symptomatic trees were PCR positive (Ct ≤ 32). However, *Ca*Las could not be detected in fruit pulp from either healthy or infected trees. Obvious phenotypic differences were found between HLB-affected and healthy fruits; symptomatic fruits were smaller and greener than the healthy fruits (Fig. 1). The soluble solids content of fruit from *Ca*Las-infected trees (9.52 g 100 g^−1^) was decreased compared with that of healthy fruit (11.24 g 100 g^−1^), whereas the acid content of fruit from *Ca*Las-infected trees (0.82%) was increased compared with that of healthy fruit (0.70%).

### Overview of the downregulated proteins in primary and secondary metabolism

**Fig. 2:**
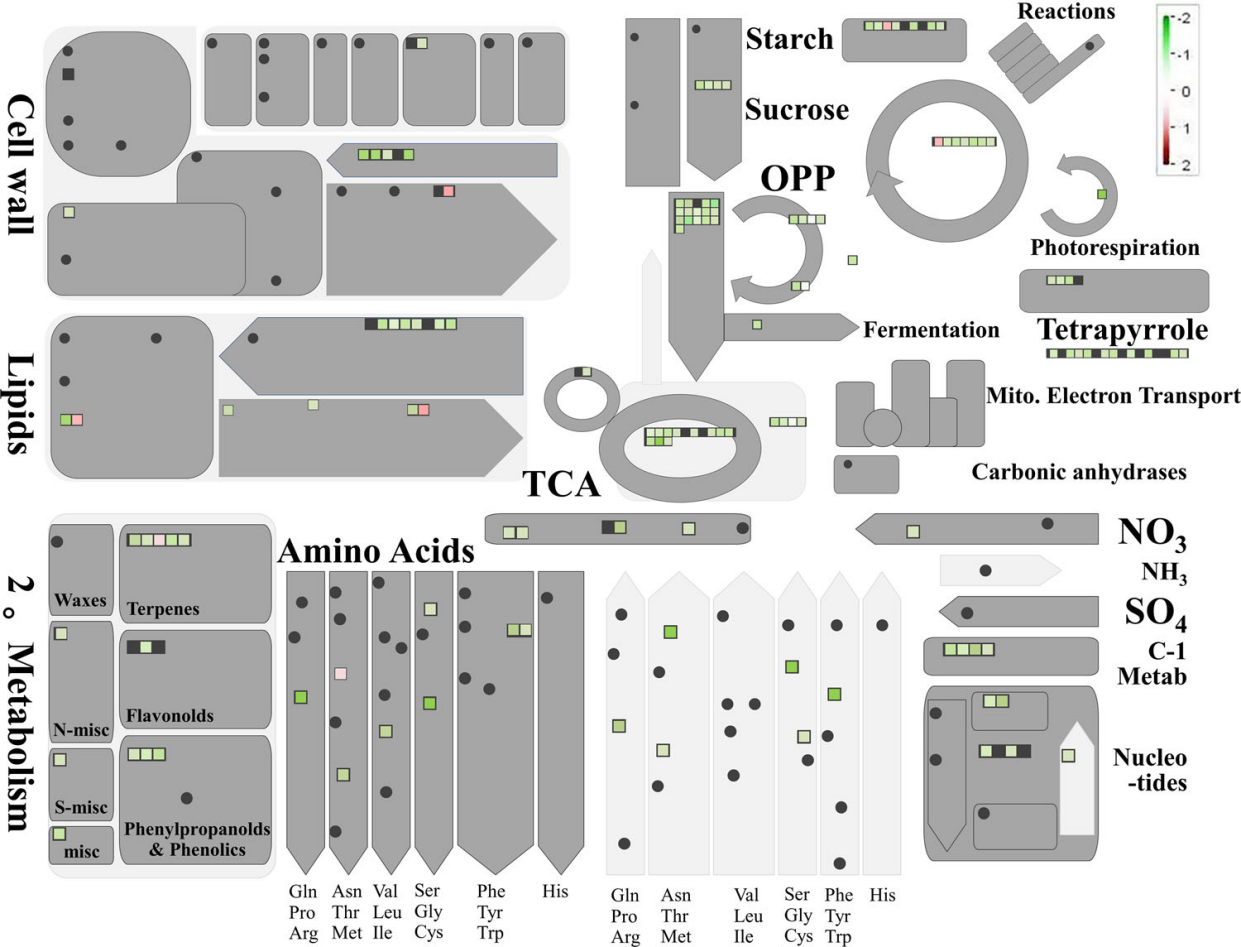
Metabolism overview in MapMan depicting differential protein expression between symptomatic and healthy fruits. Log-fold ratio was shown as a gradient of red (upregulated) and green (downregulated)

**Fig. 3:**
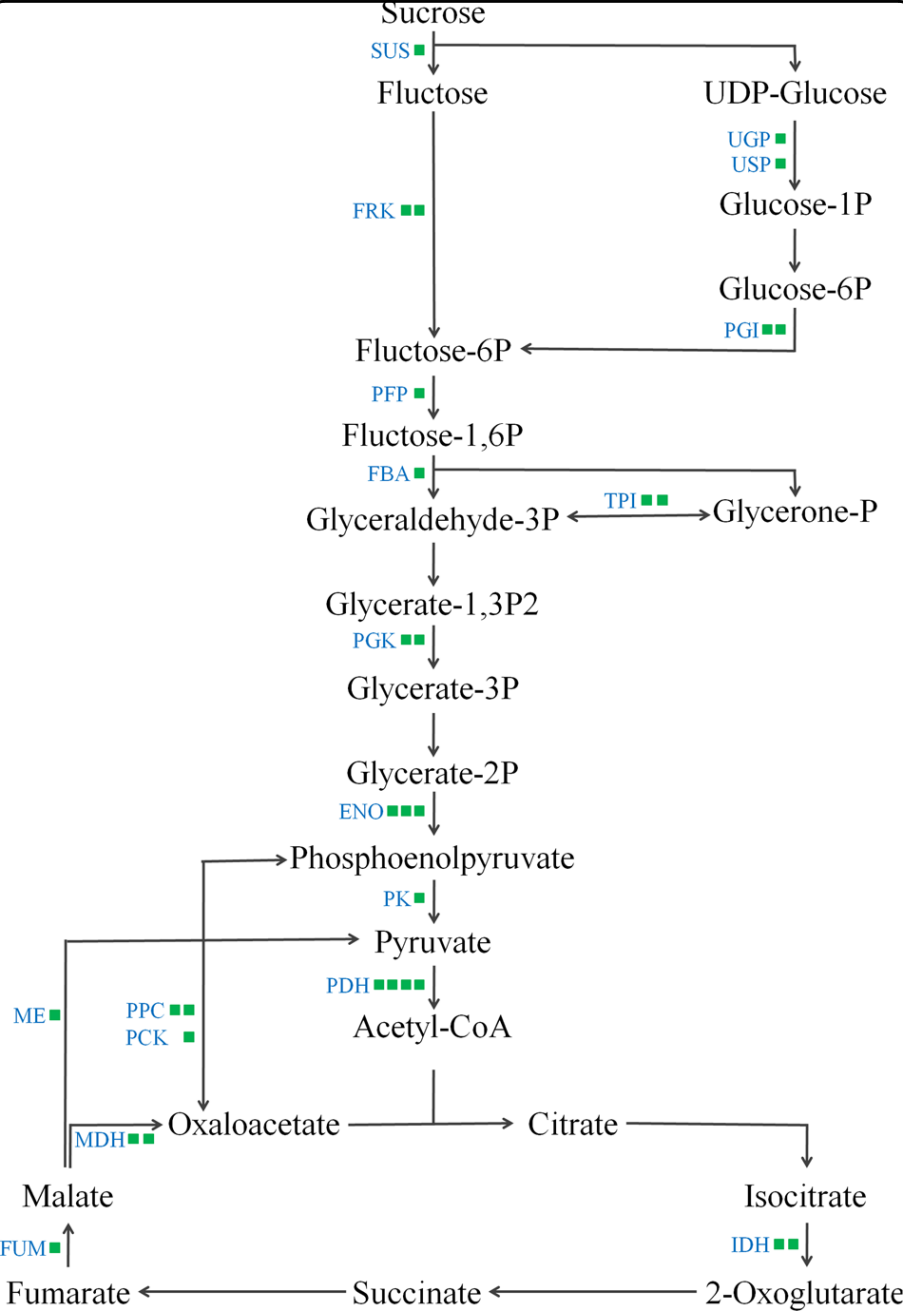
Downregulated proteins in the sugar and organic acid metabolism in HLB symptomatic fruit compared with healthy fruit. Green squares indicated the downregulated enzymes. The levels of some enzymes involved in the glyceraldehyde-3P and pyruvate pathways were decreased in the HLB-symptomatic fruit

**Fig. 4:**
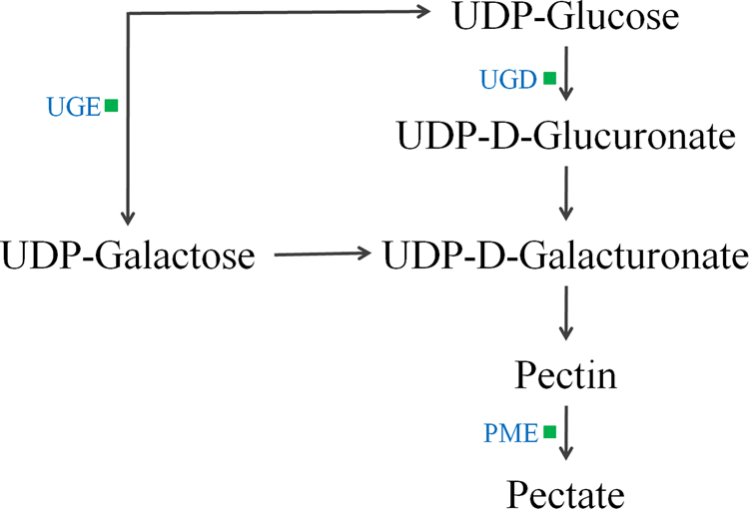
Downregulated proteins in the pectin metabolism in the HLB-symptomatic fruit compared with healthy fruit. Green squares indicated the downregulated enzymes. UGD UDP-glucose dehydrogenase, UGE UDP-d-glucose 4-epimerase, PME pectin methylesterase

**Fig. 5:**
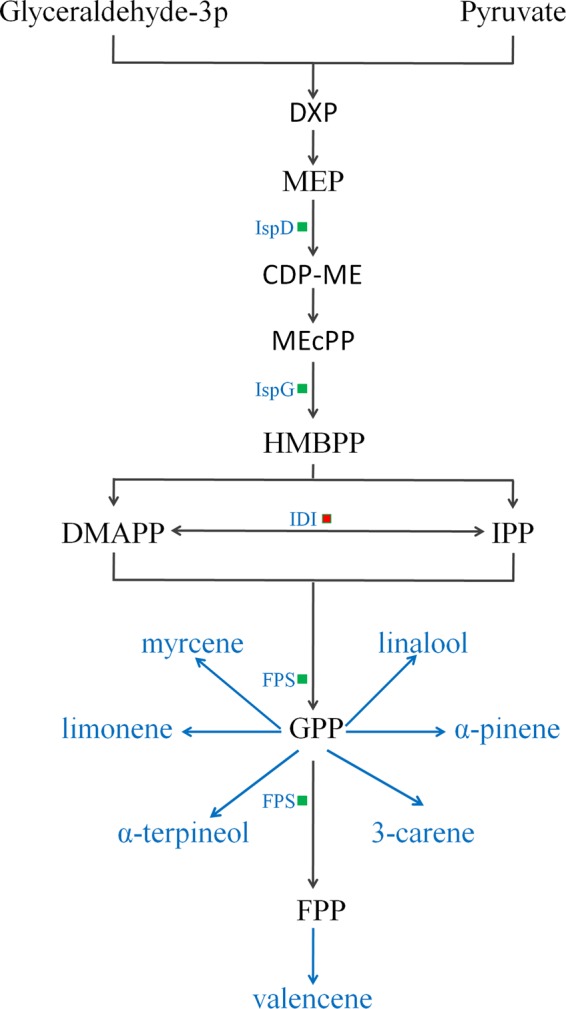
Differentially expressed monoterpene and sesquiterpene metabolism proteins in the HLB-symptomatic fruit compared with those in the healthy control. Green squares indicated the decreased enzymes. Red squares indicated the increased enzymes. DXP 1-deoxy-d-xylulose 5-phosphate, MEP 2-C-methyl-d-erythritol-4-phosphate, CDP-ME 4-diphosphocytidyl-2-C-methyl-d-erythritol, MEcPP 2-C-methyl-d-erythritol 2,4-cyclodiphosphate, HMBPP 1-hydroxy-2-methyl-2-butenyl 4-diphosphate, DMAPP dimethylallyl diphosphate, IPP isopentenyl diphosphate, GPP geranyl pyrophosphate, FPP farnesyl pyrophosphate

In total, 123 differentially expressed proteins were involved in the biological processes of sugar breakdown and glycolysis, the Calvin cycle, the tricarboxylic acid cycle (TCA), amino-acid synthesis and breakdown, UDP-sugar, terpenes, and the phenylpropanoid metabolic pathway (Fig. 2). There were 45 downregulated proteins involved in sugar and organic acid metabolism (Table 1, Fig. 3), 22 downregulated proteins involved in amino-acid metabolism (Table 2), five differentially expressed proteins involved in terpenoid metabolism (Table 3), and three downregulated proteins involved in pectin metabolism (Fig. 4). The expression of 45 enzymes involved in carbohydrate metabolism was downregulated in fruit from trees infected with *Ca*Las, including 12 oxidoreductases (EC 1 family), 12 transferases (EC 2 family), 2 hydrolases (EC 3 family), 12 lyases (EC 4 family), and 7 isomerases (EC 5 family) (Table 1). The expression of 22 enzymes involved in amino-acid metabolism was downregulated in fruit from trees infected with *Ca*Las, including eight oxidoreductases (EC 1 family), five transferases (EC 2 family), three hydrolases (EC 3 family), five lyases (EC 4 family), and one ligase (EC 6 family) (Table 2). In the terpenoid biosynthesis process (Table 3, Fig. 5), there were two downregulated oxidoreductases (EC 1 family), two downregulated transferases (EC 2 family), and one upregulated isomerase (EC 5 family) in fruit from trees infected with *Ca*Las. Regarding pectin metabolism, the downregulated proteins were pectin methylesterase (gi|641854939), UDP-d-glucose 4-epimerase (gi|568845013), and UDP-glucose dehydrogenase (gi|641853915) (Table 1, Fig. 4).

**Table 1:** Downregulated proteins involved in carbohydrate metabolism in symptomatic versus healthy Valencia orange fruit

Citrus ID	TAIR code	Protein symbol	Description	Enzyme no.	SS:HS	*P*-value
gi|568823164	AT1G54220	MTE2-3	Mitochondrial pyruvate dehydrogenase subunit 2-3	EC 2.3.1.12	0.62	1.34E–03
gi|568828917	AT2G01140	FBA3	Fructose-bisphosphate aldolase 3	EC 4.1.2.13	0.57	1.16E–04
gi|568831440	AT5G40760	G6PD6	Glucose-6-phosphate dehydrogenase 6	EC 1.1.1.49	0.52	1.55E–02
gi|568831566	AT3G01850	F28J7.18	Ribulose-phosphate 3-epimerase	EC 5.1.3.1	0.65	1.13E–02
gi|568833896	AT1G71170	F23N20.16	6-Phosphogluconate dehydrogenase-like protein	EC 1.1.1.44	0.56	1.83E–04
gi|568836722	AT4G37870	PCK1	Phosphoenolpyruvate carboxykinase 1	EC 4.1.1.49	0.58	2.19E–02
gi|568837718	AT5G52560	USP	UDP-sugar pyrophosphorylase	EC 2.7.7.64	0.47	4.50E–02
gi|568840482	AT5G38410	MXI10.13	Ribulose bisphosphate carboxylase small chain 3B	EC 4.1.1.39	0.55	9.42E–03
gi|568845013	AT4G10960	UGE5	UDP-d-glucose 4-epimerase 5	EC 5.1.3.2	0.51	2.93E–02
gi|568849997	AT3G59480	FRK3	Fructokinase 3	EC 2.7.1.4	0.55	1.02E–04
gi|568855816	AT5G03290	IDH-V	NAD^ +^ -dependent isocitric dehydrogenase V	EC 1.1.1.41	0.55	1.28E–03
gi|568856679	AT2G36530	ENO2	Enolase 2	EC 4.2.1.11	0.62	1.85E–10
gi|568864726	AT1G74030	ENO1	Enolase 1	EC 4.2.1.11	0.59	3.20E–02
gi|568868208	AT2G21170	TPI	Triosephosphate isomerase	EC 5.3.1.1	0.58	7.45E–04
gi|568870281	AT3G55440	TPI	Triosephosphate isomerase	EC 5.3.1.1	0.61	1.28E–02
gi|568870518	AT2G29560	ENOC	Cytosolic enolase 3	EC 4.2.1.11	0.64	6.79E–03
gi|568881904	AT1G56190	PGK2	Phosphoglycerate kinase	EC 2.7.2.3	0.55	8.96E–03
gi|568881906	AT1G79550	PGK3	Phosphoglycerate kinase	EC 2.7.2.3	0.55	5.80E–06
gi|568883658	AT1G65930	cICDH	Cytosolic NADP^ +^ -dependent isocitrate dehydrogenase	EC 1.1.1.42	0.58	2.47E–02
gi|572152874	AT1G53310	PPC1	Phosphoenolpyruvate carboxylase 1	EC 4.1.1.31	0.62	4.57E–04
gi|641829225	AT3G15020	mMDH2	Mitochondrial malate dehydrogenase 2	EC 1.1.1.37	0.64	1.03E–04
gi|641838381	AT5G17310	UGP2	UDP-glucose pyrophosphorylase 2	EC 2.7.7.9	0.57	2.46E–11
gi|641838649	AT1G04410	c-NAD-MDH1	Cytosolic-NAD-dependent malate dehydrogenase 1	EC 1.1.1.37	0.64	1.10E–04
gi|641839211	AT5G13420	TRA2	Transaldolase 2	EC 2.2.1.2	0.55	8.15E–04
gi|641841250	AT5G51830	FRK7	Fructokinase 7	EC 2.7.1.4	0.60	3.11E–03
gi|641841327	AT1G08110	GLYI2	Glyoxalase I2	EC 4.4.1.5	0.66	3.00E–02
gi|641842093	AT3G52990	AT3G52990	Pyruvate kinase	EC 2.7.1.40	0.60	1.30E–02
gi|641847603	AT1G67280	GLYI6	Glyoxalase I6	EC 4.4.1.5	0.66	1.70E–02
gi|641847704	AT2G19860	HXK2	Hexokinase	EC 2.7.1.1	0.64	1.68E–02
gi|641849423	AT4G35260	IDH1	NAD ^+^ -dependent isocitrate dehydrogenase 1	EC 1.1.1.41	0.57	4.29E–02
gi|641851263	AT1G68750	PPC4	Phosphoenolpyruvate carboxylase 4	EC 4.1.1.31	0.63	3.05E–02
gi|641851457	AT1G13700	PGL1	6-Phosphogluconolactonase 1	EC 3.1.1.31	0.54	3.89E–03
gi|641853224	AT1G48030	mtLPD1	Mitochondrial lipoamide dehydrogenase 1	EC 1.8.1.4	0.65	5.78E–05
gi|641853915	AT5G15490	UGD3	UDP-glucose dehydrogenase	EC 1.1.1.22	0.58	2.33E–02
gi|641854939	AT3G14310	PME3	Pectin methylesterase 3	EC 3.1.1.11	0.62	7.18E–05
gi|641859572	AT1G11840	GLYI3	Glyoxalase I3	EC 4.4.1.5	0.43	2.84E–02
gi|641862435	AT4G24620	PGI1	Plastid phospho-glucose (Glc) isomerase	EC 5.3.1.9	0.55	7.70E–04
gi|641863965	AT1G59900	E1 ALPHA	Pyruvate dehydrogenase complex E1 alpha subunit	EC 1.2.4.1	0.61	4.08E–03
gi|641864450	AT5G50850	MAB1	Pyruvate dehydrogenase E1 beta	EC 1.2.4.1	0.57	8.81E–03
gi|641865848	AT5G42740	MJB21.12	Glucose-6-phosphate isomerase	EC 5.3.1.9	0.54	4.45E–04
gi|641866581	AT1G76550	F14G6.15	Pyrophosphate:fructose-6-phosphate 1-phosphotransferase	EC 2.7.1.90	0.52	2.58E–07
gi|641867382	AT4G00570	NAD-ME2	NAD-dependent malic enzyme 2	EC 1.1.1.39	0.55	2.68E–04
gi|641867393	AT2G45790	PMM	Phosphomannomutase	EC 5.4.2.8	0.52	2.41E–03
gi|641868430	AT2G47510	FUM1	Fumarate hydratase 1	EC 4.2.1.2	0.53	7.83E–04
gi|641868466	AT4G02280	SUS3	Sucrose synthase 3	EC 2.4.1.13	0.61	4.51E–03

*SS* symptomatic fruit samples, *HS* healthy fruit samples

**Table 2:** Differentially regulated enzymes (proteins) involved in amino-acid metabolism in symptomatic versus healthy Valencia orange fruit

Citrus ID	TAIR code	Protein symbol	Description	Enzyme no.	SS:HS	*P*-value
gi|568821659	AT1G80560	IMD2	3-Isopropylmalate dehydrogenase 2	EC 1.1.1.85	0.66	3.20E–03
gi|568825237	AT5G66120	K2A18.20	Putative 3-dehydroquinate synthase	EC 4.2.3.4	0.55	2.42E–04
gi|568825398	AT2G17630	PSAT2	Pyridoxal phosphate-dependent transferase	EC 2.6.1.52	0.66	2.54E–02
gi|568867337	AT1G49820	MTK	5-Methylthioribose kinase	EC 2.7.1.100	0.63	7.61E–03
gi|568875049	AT5G52810	SARD4	NAD(P)-binding Rossmann-fold superfamily protein	EC 4.3.1.12	0.56	4.07E–02
gi|568876134	AT3G61440	CYSC1	Cysteine synthase C1	EC 2.5.1.47	0.54	1.94E–02
gi|568881821	AT1G79440	ALDH5F1	Succinate-semialdehyde dehydrogenase	EC 1.2.1.16	0.58	1.46E–02
gi|641821618	AT2G31570	GPX2	Glutathione peroxidase 2	EC 1.11.1.9	0.64	4.29E–02
gi|641823192	AT3G17760	GAD5	Glutamate decarboxylase 5	EC 4.1.1.15	0.64	4.45E–04
gi|641823815	AT4G13940	MEE58	*S*-adenosyl-l-homocysteine hydrolase	EC 3.3.1.1	0.64	1.43E–03
gi|641831913	AT5G48220	MIF21.11	Aldolase-type TIM barrel family protein	EC 4.1.1.48	0.57	1.88E–05
gi|641833609	AT4G24830	F6I7.40	Argininosuccinate synthase	EC 6.3.4.5	0.49	3.92E–02
gi|641838358	AT1G65820	F1E22.17	Putative glutathione *S*-transferase	EC 1.11.1.9	0.61	2.68E–02
gi|641841873	AT1G14810	F10B6.22	Aspartate semialdehyde dehydrogenase	EC 1.2.1.11	0.64	3.17E–02
gi|641842227	AT3G24170	GR1	Glutathione-disulphide reductase 1	EC 1.8.1.7	0.50	2.37E–02
gi|641848539	AT4G31870	GPX7	Glutathione peroxidase 7	EC 1.11.1.9	0.53	2.21E–02
gi|641849692	AT3G54640	TSA1	Alpha-tryptophan synthase	EC 4.2.1.20	0.65	3.45E–02
gi|641855678	AT5G43850	ARD4	Acireductone dioxygenase 4	EC 1.13.11.54	0.65	3.13E–02
gi|641859599	AT1G11860	F12F1.30	Aminomethyltransferase	EC 2.1.2.10	0.48	3.54E–02
gi|641859748	AT1G12050	FAH	Putative fumarylacetoacetase	EC 3.7.1.2	0.54	6.07E–04
gi|641862021	AT1G44820	T12C22.9	Peptidase M20/M25/M40 family protein	EC 3.5.1.14	0.57	2.13E–02
gi|641862593	AT5G26780	SHM2	Serine hydroxymethyltransferase 2	EC 2.1.2.1	0.57	2.13E–02

*SS* symptomatic fruit samples, *HS* healthy fruit samples

**Table 3:** Differentially regulated enzymes (proteins) involved in terpenoid metabolism in symptomatic versus healthy Valencia orange fruit

Citrus ID	TAIR code	Protein symbol	Protein description	Enzyme no.	SS:HS	*P*-value
gi|568833221	AT2G02500	IspD	2-C-methyl-d-erythritol-4-phosphate cytidylyltransferase	EC 2.7.7.60	0.62	3.19E–03
gi|568858669	AT3G63520	CCD1	9-Cis-epoxycarotenoid dioxygenase	EC 1.13.11.51	0.57	4.05E–02
gi|568869804	AT5G60600	IspG	Hydroxy-2-methyl-2-butenyl 4-diphosphate (HMBPP) synthase	EC 1.17.7.1	0.63	8.95E–03
gi|641835071	AT5G16440	IDI1	Isopentenyl diphosphate delta-isomerase I	EC 5.3.3.2	1.69	2.78E–05
gi|641840281	AT4G17190	FPS2	Farnesyl diphosphate synthase	EC 2.5.1.10	0.57	6.89E–05

*SS* symptomatic fruit samples, *HS* healthy fruit samples

### Downregulated proteins involved in sugar and organic acid metabolism and homeostasis

Most of the proteins included in sucrose degradation and glycolytic pathways were downregulated when compared with those in healthy mature fruit. Sucrose is degraded by sucrose synthase (SUS) to generate fructose and UDP-glucose. The expression of SUS (gi|641868466) was decreased in fruit from trees infected with *Ca*Las. Fructokinase (gi|568849997 and gi|641841250) and pyrophosphate:fructose-6-phosphate 1-phosphotransferase (gi|641866581), which catalyse the phosphorylation of fructose, were also reduced on the protein level in symptomatic fruits. UDP-glucose can be converted into glucose-1P catalysed by UDP-glucose pyrophosphorylase (gi|641838381) and UDP-sugar pyrophosphorylase (gi|568837718), which is involved in the glycolysis process (Fig. 3). It can also be converted into UDP-glucuronate catalysed by UDP-glucose dehydrogenase (gi|641853915), which is involved in the synthesis of lignin (Fig. 4). Three of the enzymes were found at lower levels in diseased fruit than in the healthy control. All homologous proteins of Arabidopsis enolases (gi|568856679, gi|568864726, and gi|568870518) and phosphoglycerate kinases (gi|568881904 and gi|568881906) related to glycolysis were downregulated in the symptomatic fruit. Two isocitrate dehydrogenases (gi|568855816 and gi|641849423), two malate dehydrogenases (gi|641829225 and gi|641838649), and a fumarate hydratase (gi|641868430) were found at lower levels in the HLB-affected orange fruits than in the healthy control.

Conversely, many proteins associated with pyruvate metabolism were downregulated by *Ca*Las. Levels of phosphoenolpyruvate carboxylase (gi|572152874 and gi|641851263) and carboxykinase (gi|568836722), which are associated with the transformation between phosphoenolpyruvate and oxaloacetate, were decreased. Levels of malic enzyme (gi|641867382), which transforms malate to pyruvate, were reduced in fruit from *Ca*Las-infected trees. The pyruvate dehydrogenase enzyme complex connects glycolysis with the TCA cycle. Levels of two of the pyruvate dehydrogenase complex proteins (gi|641863965 and gi|641864450) were reduced, and levels of two other components of the same complex, dihydrolipoamide S-acetyltransferase (gi|568823164) and dihydrolipoamide dehydrogenase (gi|641853224), were downregulated in symptomatic fruit (Table 1).

### Changes in sugar, organic acid, and amino-acid metabolism

The standard products for 8 distinctive sugars, 5 organic acids, and 12 amino acids were run (Table 4). Some tetramethyl silane (TMS) derivatives of the non-volatile products could not be detected using GC-MS, including mannose, rhamnose, sorbitol, oxalic acid, tartaric acid, arginine, cysteine, glutamine, histidine, isoleucine, lysine, methionine, tyrosine, and cystine. Sucrose, fructose, and glucose were the main distinctive sugars in the Valencia pulp, and their concentration was decreased 26%, 15%, and 20%, respectively, in symptomatic sweet orange fruit compared with the healthy control. Although some low-content monosaccharides such as arabinose (18.88%), mannitol (38.07%), and ribose (28.79%), were increased, the main organic acids, citric acid and malic acid, decreased 6.50% and 9.20%, respectively, compared with the healthy control. The low-content organic acids, fumaric acid, quinic acid, and succinic acid, increased 48.06%, 20.03%, and 54.82%, respectively, compared with the healthy control. The levels of two acidic amino acids, aspartic acid (3.23%) and glutamic acid (13.71%), were decreased in symptomatic fruit. The following neutral amino acids were more highly accumulated in symptomatic fruit: alanine (10.00%), asparagine (128.12%), glycine (31.58%), leucine (73.66%), phenylalanine (79.44%), proline (20.57%), serine (74.14%), threonine (217.79%), tryptophan (61.83%), and valine (20.39%).

**Table 4:** Non-volatile compound content in symptomatic and healthy Valencia orange fruit

TMS derivative (µg g^–1^)	Rt (min)	Ion model (m/z)	Healthy fruits	Symptomatic fruits	*P*-value
Sugars					
Arabinose (TMS)_4_	29.37	307	1.99 ± 0.39	2.36 ± 1.24	6.44E–01
Fructose (TMS)_5_	34.87/35.13	307	344.81 ± 14.02	290.44 ± 35.07	6.73E–02
Glucose (TMS)_5_	35.47/35.85	319	263.22 ± 10.38	210.59 ± 20.79	1.72E–02
Mannitol (TMS)_6_	36.21	319	1.33 ± 0.13	1.84 ± 0.78	3.31E–01
Myo-inositol (TMS)_6_	39.81	305	102.25 ± 6.45	96.87 ± 5.12	3.21E–01
Ribose (TMS)_4_	29.79	307	0.42 ± 0.03	0.54 ± 0.11	1.25E–01
Sucrose (TMS)_8_	51.29	361	379.52 ± 24.37	277.33 ± 47.93	3.02E–02
l-Xylose (TMS)_4_	29.22	307	3.36 ± 0.58	2.95 ± 0.14	3.01E–01
Organic acids					
Citric acid (TMS)_4_	33.28	273	144.80 ± 8.10	135.38 ± 11.97	3.60E–01
Fumaric acid (TMS)_2_	19.12	245	0.07 ± 0.01	0.10 ± 0.00	3.54E–03
Malic acid (TMS)_3_	23.96	245	66.29 ± 9.75	60.19 ± 3.14	3.61E–01
Quinic acid (TMS)_5_	34.45	345	13.91 ± 0.69	16.69 ± 0.28	2.99E–03
Succinic acid (TMS)_2_	18.08	247	0.32 ± 0.04	0.49 ± 0.13	8.30E–02
Amino acids					
d-Alanine (TMS)_2_	10.55	116	0.74 ± 0.07	0.82 ± 0.05	1.94E–01
l-Asparagine (TMS)_3_	29.13	232	7.29 ± 2.94	16.62 ± 12.73	2.84E–01
l-Aspartic acid (TMS)_3_	24.89	188	9.29 ± 2.38	8.99 ± 8.23	9.55E–01
d-Glutamic acid (TMS)_3_	27.70	246	3.45 ± 0.61	2.98 ± 1.47	6.34E–01
Glycine (TMS)_3_	17.83	174	0.91 ± 0.09	1.20 ± 0.25	1.39E–01
l-Leucine (TMS)_2_	16.75	158	0.04 ± 0.01	0.07 ± 0.02	1.07E–01
l-Phenylalanine (TMS)_2_	27.64	218	0.71 ± 0.14	1.28 ± 0.59	1.80E–01
l-Proline (TMS)_2_	17.43	142	6.51 ± 1.21	7.84 ± 2.16	4.03E–01
l-Serine (TMS)_3_	19.91	204	0.76 ± 0.03	1.32 ± 0.36	5.31E–02
l-Threonine (TMS)_3_	20.78	101	0.06 ± 0.00	0.19 ± 0.08	5.84E–02
l-Tryptophan (TMS)_3_	42.15	202	0.18 ± 0.05	0.30 ± 0.20	3.85E–01
l-Valine (TMS)_2_	14.72	144	0.25 ± 0.03	0.30 ± 0.04	1.56E–01

*Rt* retention time

### Low levels of terpenoids and proteins involved in terpenoid metabolism

According to MS peak identification and standard confirmation, six monoterpenoids and one sesquiterpenoid (valencene) were identified in the Valencia pulp. All levels were lower in the symptomatic fruits than those in the healthy fruits: α-pinene (77.33%), 3-carene (87.20%), myrcene (85.53%), limonene (82.29%), linalool (77.57%), α-terpineol (72.16%), and ( + )-valencene (78.66%) (Table 5).

**Table 5:** Monoterpenoids and valencene content in symptomatic and healthy Valencia orange fruit

Concentration (µg g^–1^)	RI	Healthy fruits	Symptomatic fruits	*P*-value
( + )-Valencene	1023.20	1248.06 ± 486.29	266.31 ± 67.67	1.06E–01
3-Carene	1156.40	25.17 ± 0.04	3.22 ± 0.36	1.35E–04
Limonene	1202.64	42,458.08 ± 8754.63	7519.50 ± 2432.43	3.22E–02
Linalool	1528.46	187.30 ± 30.2	42.02 ± 22.22	3.17E–02
Myrcene	1539.75	854.79 ± 94.58	123.69 ± 44.08	1.00E–02
α-Pinene	1678.32	240.56 ± 80.62	54.54 ± 3.86	8.26E–02
α-Terpineol	1710.00	120.47 ± 21.85	33.53 ± 2.20	3.04E–02

*RI* retention index

All isoprenoids are synthesised from isopentenyl diphosphate (IPP) and dimethylallyl diphosphate (DMAPP), the two building blocks, which are produced by the mevalonic acid (MVA) and 2-C-methyl-d-erythritol-4-phosphate (MEP) pathways. 2-C-methyl-d-erythritol-4-phosphate cytidylyltransferase (IspD) catalyses MEP to form 4-diphosphocytidyl-2-C-methyl-d-erythritol (CDP-ME) and is the third enzyme of the MEP pathway. Hydroxy-2-methyl-2-(E)-butenyl 4-diphosphate synthase (IspG) catalyses 2-C-methyl-d-erythritol 2,4-cyclodiphosphate (MEcPP) to form 1-hydroxy-2-methyl-2-butenyl 4-diphosphate (HMBPP). That is the penultimate catalysing step of the biosynthesis of IPP and DMAPP through the MEP pathway. The enzyme ispD and ispG (gi|568833221 and gi|568869804) were all downregulated in symptomatic fruit. The levels of farnesyl diphosphate synthase (gi|641840281), which catalyses the rate-limiting step in geranyl pyrophosphate (GPP) and farnesyl pyrophosphate (FPP) biosynthesis, was also reduced in symptomatic fruit (Fig. 5).

## Discussion

A total of 123 differentially expressed proteins were found in primary and secondary metabolism pathways, when comparing HLB-affected fruit with healthy fruit; the fold change threshold was designated as upregulation/downregulation = 1.5/0.67. The 1.5-fold change has been used as a standard for iTRAQ-based quantitative proteomic analysis in tetraploid wheat (*Triticum turgidum*)^18^ and *Flammulina velutipes*^19^, two examples where changes in protein expression were mostly <2-fold. From a purely technical point of view, it may be thought as a limitation of iTRAQ because of the fold changes in iTRAQ were typically <2. However, iTRAQ for quantitative proteomics could provide accurate quantification spanning two orders of magnitude^20^, and the value with false discovery rate < 0.05 was considered authentic. Although proteins represent the products translated from the transcriptome, protein expression depends only in part on the levels of gene expression.

### Host proteins involved in primary metabolism were downregulated  in fruit from *Ca*Las-infected trees

Glycolysis is a central and important metabolic pathway in plants and can provide energy, reducing power, and pyruvate to fuel the TCA cycle, and precursors for amino acid, fatty acid and secondary metabolite biosynthesis^21^. Most of the glycolysis enzymes are upregulated during the development of citrus fruit from the fruit enlargement stage into the fruit maturation and ripening stages^22^. Previous reports have also revealed that the plants in which the glycolysis enzymes were downregulated show significantly retarded growth^23–25^. For *Ca*Las-infected citrus, proteins involved in glycolysis are downregulated in the symptomatic fruits compared with those in healthy fruits^1,12,16^. In our study, 17 proteins involved in the glycolysis pathway were downregulated in orange fruits by *Ca*Las, including two PGKs (gi|568881904 and gi|568881906) and one PFP (gi|641866581). The downregulation of glycolysis proteins in HLB-affected oranges was likely responsible for production of the smaller and unripened fruits.

### Non-volatile and volatile compounds were differentially regulated in fruit from *Ca*Las-infected trees

When citrus is affected by HLB, Brix and Brix/acid levels are decreased, and the fruit flavour is changed. °Brix is made up of sugars, primarily sucrose, fructose, and glucose. In this study, we found that the contents of sucrose and glucose in the pulp of HLB-symptomatic fruit were decreased significantly when compared with those of the healthy fruit, and the content of fructose and myo-inositol also showed a downward trend. The results corroborated the conclusion that the sugar content is reduced in fruit from *Ca*Las-infected trees^4,6,7^. At the same time, we also detected that the content of arabinose, mannitol and ribose in symptomatic fruit increased slightly compared with that in healthy fruit. In symptomatic fruit, malic acid levels were decreased, whereas fumaric acid and succinic levels were increased, which was consistent with the report of Sliz et al.^7^. Citric acid, the dominant organic acid of citrus fruit, was decreased in symptomatic fruit. This finding was similar to the result of Sliz et al.^7^, in which they found that the content of citric acid was decreased in asymptomatic fruit from *Ca*Las-infected trees. Chin et al.^6^ found that quinic acid was increased in symptomatic fruit. We also found that the content of quinic acid, lower than that of citric acid and malic acid, was present at higher levels in the symptomatic fruit when compared with the levels in healthy fruit.

Volatile compounds are produced in oil glands of the peels, leaves, and other citrus plant parts^26^. Combining both mass spectra and retention index value matching, seven terpenes and terpene alcohols were identified. Valencene, a kind of sesquiterpene, is the second most abundant terpene after limonene in orange juice and has been used for many years as a quality and maturity marker^27^. As shown in Table 5, concentration of valencene was lower in symptomatic fruit than in control fruit. This suggests the symptomatic fruit had not matured in a normal way, similar to other reports about the valencene concentrations in HLB-affected orange juice^4,8^. However, the deduced concentration of monoterpenes in the symptomatic fruit pulp was different than that found in other studies. Levels of terpenes, such as linalool, were increased in the orange juice of symptomatic fruit in the published literatures^4,8^. Kiefl et al.^8^ reported that the juice oil levels were similar in HLB-affected orange juice (174 mg kg^–1^) and healthy control orange juice (185 mg kg^–1^). They also found *Ca*Las infection induces significantly higher levels of peel oil aroma volatiles. Some kinds of volatile biomolecules, such as (Z)-4-ecenal and (E,E)-2,4-decadienal, could pass into the juice from the peel, and increase concentrations in the orange juice by 38 and 19%, respectively^28,29^. This indicated that in the process of squeezing juice, the increased volatiles in HLB-affected peel oil were transferred into the juice and might change the concentration of volatiles in juice. In our study, we removed the peel and measured the monoterpene concentration in the fruit pulp through GC-MS and found that they were reduced in the juice sacs of symptomatic fruits. The output of healthy orange juice with commercially acceptable quality has been decreasing year by year since HLB has attacked the Florida citrus industry. It may be possible to utilise some of the volatile aromatic metabolites found in healthy fruit peel to improve the flavour of processed orange juice.

### Correlation between the downregulation of proteins and decreased terpenoid levels

Studies on the pathogenic mechanism of HLB have shown that *Ca*Las infection induces the production of callose deposition in plants, causing the obstruction of phloem and impeding the transport of organic compounds from source to sink, which results in starch deposition and mottled leaves^30,31^. In citrus, sucrose is translocated to the fruits from the leaves throughout fruit development and constitutes approximately 50% of the total soluble sugar^32^. The significantly reduced sucrose in the symptomatic fruits might be the result of a block from the leaves (source) into the fruits (sink) induced by the *Ca*Las infection^15,30^. Imported sucrose in sink organs can be degraded to fructose and glucose by invertase or to fructose and UDP-glucose by SUS. We did not find change in the levels of invertase. However, an SUS, SUS3 (gi|641868466), was downregulated in the symptomatic fruits compared with healthy fruits. SUS3 expression was confirmed in the sink tissues of Arabidopsis^33^. Interestingly, fructose content in the same fruits was reduced because of the decreased levels of sucrose and downregulated SUS (Table 4).

Glyceraldehydes-3-P and pyruvate were the intermediate products in the glycolysis process, providing the carbon skeleton for terpenoid compound biosynthesis in the fruit. In this process, six proteins catalyzing the transformation from glucose and fructose to glyceraldehyde-3-P were present at lower levels in the HLB-symptomatic fruits than in the healthy fruits (Fig. 3). Two PGIs (gi|641862435 and gi|641865848) transformed glucose-6P to fluctose-6P, two FRKs (gi|568849997 and gi|641841250) phosphorylated fructose, PFP (gi|641866581) and FBA (gi|568828917) catalysed fructose-6P to glyceraldehyde-3P. Pyruvate was obtained from dephosphorylated phosphoenolpyruvate through PK (gi|641842093) and from a non-TCA-cyclic flux compound, malate, via ME (gi|641867382) catalysis. The pyruvate precursor, phosphoenolpyruvate, was obtained from glycerate-2P catalysed by enolases (gi|568856679, gi|568864726, and gi|568870518) and from oxaloacetate catalysed by PCK (gi|568836722). The levels of these proteins associated with pyruvate metabolism were decreased in the HLB-symptomatic fruit compared with those in the healthy fruit (Fig. 3). Symptomatic fruit was similar to immature fruit, and the PCK and ME increase in healthy fruit agreed with the determination that these proteins accumulate during fruit development and ripening^22^.

Terpenoids have many volatile representatives and compose the largest group of plant secondary metabolic compounds. IPP and its allylic isomer DMAPP are the five universal carbon precursors for all terpenoid products. In the plastids, IPP is synthesised from pyruvate and glyceraldehyde-3-phosphate via the MEP pathway, in which there are seven subsequent enzymatic steps. The third enzyme, IspD (gi|568833221), and sixth enzyme, IspG (gi|568869804), involved in the conversion of MEP into CDP-ME, and MEcPP into HMBPP, respectively^34^, were downregulated in the symptomatic fruit (Fig. 5). IspG overexpression could lead to accumulation of intermediate HMBPP^35^. The reduced levels of IspD and IspG in HLB fruit might affect the production of HMBPP.

IDI1 (type I IPP isomerase) is mainly found in the plastid and converts IPP to DMAPP^36^. Pankratov et al.^37^ determined that mutations of IDI1 in tomato could reduce overall carotenoid accumulation in fruits. Carotene cleavage dioxygenases (CCD) catalyse the cleavage of carotenoids to apocarotenoids. Overexpression of AtCCD1 could induce β-ionone emission^38^, whereas CCD1 loss-of-function mutants in Arabidopsis showed an increase in their seed carotenoid levels^39^. IDI1 (gi|641835071) was upregulated, and CCD1 (gi|568858669) (Table 3) was downregulated in the symptomatic orange fruit, which might lead to carotenoid accumulation and terpenoids reduction in the fruit pulp. *Ca*Las appears to accelerate the biosynthesis of carotenoid pigments in HLB-affected Valencia sweet orange leaves^40^.

FPS catalyses consecutive *E*-condensations of two IPPs with one DMAPP to form the intermediate GPP, then form the end product FPP. GPP is catalysed to form an array of monoterpenes^41^, and FPP is catalysed to form sesquiterpenes and di-, tri-, and tetra-terpenes in high to low organisms. FPS is considered as a rate-limiting enzyme that determines the flow rate of GPP and FPP^42^. Overexpression of the FPS2 protein could increase terpenoid accumulation in plants^43^. FPS2 (gi|641840281) was downregulated in the HLB-symptomatic fruit, and the concentration of monoterpenes and valencene were decreased in the pulp of these fruits compared with that of healthy controls.

## Conclusions

The results from studies performed on citrus fruit pulp from *Ca*Las-infected trees revealed significant suppression and metabolic dysfunction in sugar and organic acid metabolism and homeostasis, and terpenoid metabolism. These cause major disruptions in the production of proteins, sugars, organic acids, and volatiles, resulting in lower levels of sugars and terpenoids in symptomatic fruit pulp, and thereby causes many of the negative attributes found in juice produced from such fruit.

## Materials and methods

### Materials

The harvest season for Valencia orange covers 4 months, typically from late-February or early-March, to late-May or early-June. Fruit harvested in the middle to late months have the highest concentrations of many important aroma compounds, compared with those harvested in the very early or late months of the Valencia maturity season^44^. Fruits of 8-year-old Valencia orange (*Citrus sinensi*s) scions on Swingle citrumelo rootstocks were harvested from a commercial citrus grove in St. Cloud, Florida, USA, on 1 April 2015. Because of the widespread incursion of HLB in Florida and in this specific grove as well, we found only two PCR-negative and healthy trees. Consequently, biological sample replication was restricted to just two healthy trees to use as controls, for comparison with three replicates of symptomatic trees. Symptomatic and healthy fruits were harvested from PCR-positive and PCR-negative trees, respectively. Five fruits randomly selected from one tree were peeled, and pulp was mixed as one biological replicate. The peeled pulp of fruits was stored at –80℃.

Analytical grade standards of non-volatile polar compounds; volatiles; internal standards, including 4-heptadecanone and ribitol, n-alkanes (C8–C20) and chemical reagents; and dichloromethane, pyridine, methoxyamine hydrochloride (MEOX) and *N*-methyl-*N*-(trimethylsilyl)-trifluoroacetamide (MSTFA) were purchased from Sigma (Sigma-Aldrich Co., St. Louis, MO, USA).

### Protein extraction

Total protein was extracted using a phenol method^45^. A total of 5 g of frozen juice sac samples were ground in liquid nitrogen, and 12 mL of protein extraction buffer (0.5 M Tris (pH 7.5), 0.1 M KCl, 0.7 M sucrose, 0.05 M EDTA, 0.05 M dithiothreitol, 1 mM phenylmethanesulfonyl fluoride, 1% 2-mercaptoethanol, and 0.1% protease inhibitor mix and 18 mL Tris-saturated phenol) was added. After three more rounds of phenol extraction, the top phenol phase was moved into a new tube and 30 mL of ammonium acetate methanol solution was poured into the tube to precipitate the pellet. The pellet was formed by centrifugation and washed twice, using 10 mL ice cold 100% methanol for one wash and ice cold 100% acetone for the second wash.

### Proteomic iTRAQ and data analysis

For the iTRAQ experiments, five specimens were labelled and analysed (three biological replicates of the symptomatic sample and two biological replicates of the healthy sample). The procedutres were performed according to the manufacturer’s protocols at the Interdisciplinary Center for Biotechnology Research (ICBR) of the University of Florida (Gainesville, FL, USA). For protein identification, the ProteinPilot v5.0 software integrated with Paragon™ algorithm (https://sciex.com/products/software/proteinpilot-software) was employed against the GenBank subset of *C. sinensis* plants in the FASTA formation (downloaded in December 2012), using the original MS/MS data for database searching. The statistical analysis for relative quantification of proteins was performed using the Pro Group™ Algorithm embedded in the ProteinPilot Software. A differentially expressed protein had to be quantified with a fold change >1.5 or <0.67 and a *P*-value <0.05. Arabidopsis orthologues were determined for each differentially expressed protein through local blastx (*e*-value < 10^−3^) to the predicted Arabidopsis proteins in the TAIR database (TAIR10_pep_20101028). The biological interpretations of the relatively quantified proteins were obtained by attributing them to their own metabolic pathways with Kyoto Encyclopaedia of Genes and Genomes (KEGG) annotation and MAPMAN 3.6.0RC1 software^46^.

### Preparation of the aroma isolates

A total of 50 g of pulp frozen in liquid nitrogen was ground and transferred into a plastic bottle; 100 mL of distilled dichloromethane was added, and the pulp powder was extracted twice. The supernatant was filtered using filter paper, and 250 μL of 2 mg mL^–1^ 4-heptadecanone was added. The volatile compounds were isolated using the solvent-assisted flavour evaporation extraction method and concentrated to 150 μL according to Huang’s description^47^.

### Preparation and TMS derivatization of non-volatile metabolites

Non-volatile organic compounds were extracted and derivatized according to Lisec’s protocol^48^, although several modifications were made. A total of 20 g pulp frozen in liquid nitrogen was ground to powder and transferred to a plastic bottle, and 60 mL of 100% distilled methanol (precooled at –20 °C) was added into the bottle and vortexed for 10 s; then, 1 mL of 10 mg mL^−1^ ribitol was added as an internal quantitative standard. The bottle was shaken for 30 min at 70 °C and centrifuged for 10 min at 11,000 *g* at room temperature. The upper phase was moved to a new glass bottle and evaporated to remove water using a rotary evaporator and exhaustively dried under vacuum conditions using a freeze dryer (Labconco FreeZone 2.5, Kansas City, MO, USA). A total of 10 mg freeze-dried powder was added, along with 80 µL of methoxyamination reagent, to the aliquots; 40 µL of the solution was transferred into a new tube with another 40 µL of methoxyamination reagent and shaken for 2 h at 37 °C. A total of 70 µL MSTFA solution was added to the sample aliquots and shaken for 30 min at 37 °C again. Then, the liquid was filtered and transferred into glass vials and run on the GC for no >24 h.

### GC-MS analysis of volatile compounds

GC was performed on a PerkinElmer Clarus 680 (PerkinElmer, Inc., MA, USA) equipped with an autosampler. A trace-free fatty acid phase column (30 m × 0.25 mm, 0.25 μm film thickness) was used. MS analysis was performed on a PerkinElmer Clarus SW8T (PerkinElmer).

Helium carrier gas was used at a flow rate (constant mode) of 5.0 mL min^−1^. The oven temperature ramp for the GC analysis of volatile compounds was as follows: the initial oven temperature was 40 °C, held for 1 min; then heating at 30 °C min^–1^ up to 100 °C, held for 2 min; at the end heating at 5 °C min^–1^ up to 230 °C, held for 10 min. It took 41 min for the entire run.

### GC-MS analysis for TMS derivatives

GC-MS analysis was performed on an Agilent GC-MS 5975C-7890A (Agilent Technologies, CA, USA) with an autosampler (7693). A Rxi-5 MS column (Crossbond diphenyl dimethyl polysiloxane; 30 m × 0.25 mm, 0.25 μm film thickness) from Restek (Cat#:13423, Restek Corporation, Bellefonte, PA, USA) was used.

The carrier gas, helium, ran at a flow rate (constant mode) of 1.1 mL min^–1^. In all, 1 µL of TMS derivatives was injected into an injector with temperature 230 °C and split ratio 10:1. The oven temperature ramp for the GC analysis of volatile compounds was as follows: initial temperature 70 °C, held for 5 min; then heating at 4 °C min^–1^ to 270 °C, at the end 20 °C min^–1^ to 320 °C for 4.5 min. It took 62 min for the entire run. The fixed MS settings were scan mode for the analysis of the TMS derivatives, full with a mass range m/z 60–650.

### MS peak identification and internal standard quantification

GC-MS chromatograms were analysed using TurboMass software version 5.4.2 (PerkinElmer, Waltham, MA, USA). Peaks were first identified by comparing their mass spectra with the NIST library (National Institute of Standards and Technology, Gaithersberg, MA, USA) and 9th edition Wiley (John Wiley and Sons, Inc., Hoboken, NJ, USA). Identified results from libraries were further confirmed, after comparing the retention time and mass spectra of derivatized standards with those of the detected chemical compounds. 4-Heptadecanone was used as an internal standard for the analysis of volatile compounds, and ribitol acted as an internal standard for non-volatile compounds. Semiquantitative measurements were implemented for the metabolite concentrations.

### Statistical analysis of metabolic compounds

Pooled samples from five randomly selected fruits were used for each individual biological replicated tree. The differences of metabolic compounds between healthy and symptomatic fruits were examined by an analysis of variance using the JMP 12.0 statistical package (SAS Institute, Cary, NC, USA). A two-sample *t*-test was performed to assess the significance of differences for specific metabolic compounds between healthy and symptomatic fruits.
